# Identifying perinatal self-harm in electronic healthcare records using natural language processing

**DOI:** 10.1192/bjo.2021.74

**Published:** 2021-06-18

**Authors:** Karyn Ayre, Andre Bittar, Rina Dutta, Somain Verma, Joyce Kam

**Affiliations:** 1 Section of Women's Mental Health, Health Services and Population Research Department, Institute of Psychiatry, Psychology and Neuroscience, King's College London, South London and Maudsley NHS Foundation Trust; 2 Academic Department of Psychological Medicine, Institute of Psychiatry, Psychology and Neuroscience, King's College London; 3 South London and Maudsley NHS Foundation Trust, Academic Department of Psychological Medicine, Institute of Psychiatry, Psychology and Neuroscience, King's College London; 4 King's College London GKT School of Medical Education

## Abstract

**Aims:**

1.To generate a Natural Language Processing (NLP) application that can identify mentions of perinatal self-harm among electronic healthcare records (EHRs)

2.To use this application to estimate the prevalence of perinatal self-harm within a data-linkage cohort of women accessing secondary mental healthcare during the perinatal period.

**Method:**

Data source: the Clinical Record Interactive Search system. This is a database of de-identified EHRs of secondary mental healthcare service-users at South London and Maudsley NHS Foundation Trust (SLaM). CRIS has pre-existing ethical approval via the Oxfordshire Research Ethics Committee C (ref 18/SC/0372) and this project was approved by the CRIS Oversight Committee (16-069). After developing a list of synonyms for self-harm and piloting coding rules, a gold standard dataset of EHRs was manually coded using Extensible Human Oracle Suite of Tools (eHOST) software. An NLP application to detect perinatal self-harm was then developed using several layers of linguistic processing based on the spaCy NLP library for Python. Evaluation of mention-level performance was done according to the attributes of mentions the application was designed to identify (span, status, temporality and polarity), by comparing application performance against the gold standard dataset. Performance was described as precision, recall, F-score and Cohen's kappa. Most service-users had more than one EHR in their period of perinatal service use. Performance was therefore also measured at “service-user level” with additional performance metrics of likelihood ratios and post-test probabilities. Linkage with the Hospital Episode Statistics datacase allowed creation of a cohort of women who accessed SLaM during the perinatal period. By deploying the application on the EHRs of the women in the cohort, we were able to estimate the prevalence of perinatal self-harm.

**Result:**

Mention-level performance: micro-averaged F-score, precision and recall for span, polarity and temporality all >0.8. Kappa for status 0.68, temporality 0.62, polarity 0.91. Service-user level performance: F-score, precision, recall all 0.69, overall F-score 0.81, positive likelihood ratio 9.4 (4.8–19), post-test probability 68.9% (95%CI 53–82).

Cohort prevalence of self-harm in pregnancy was 15.3% (95% CI 14.3–16.3); self-harm in the postnatal year was 19.7% (95% CI 18.6–20.8). Only a very small proportion of women self-harmed in both pregnancy and the postnatal year (3.9%, 95% CI 3.3–4.4).

**Conclusion:**

NLP can be used to identify perinatal self-harm within EHRs. The hardest attribute to classify was temporality. This is in line with the wider literature indicating temporality as a notoriously difficult problem in NLP. As a result, the application probably over-estimates prevalence, to a degree. However, overall performance, given the difficulty of the task, is good.

Bearing in mind the limitations, our findings suggest that self-harm is likely to be relatively common in women accessing secondary mental healthcare during the perinatal period.

*Funding:* KA is funded by a National Institute for Health Research Doctoral Research Fellowship (NIHR-DRF-2016-09-042). The views expressed are those of the authors and not necessarily those of the NHS, the NIHR or the Department of Health and Social Care. RD is funded by a Clinician Scientist Fellowship (research project e-HOST-IT) from the Health Foundation in partnership with the Academy of Medical Sciences which also party funds AB. AB's work was also part supported by Health Data Research UK, an initiative funded by UK Research and Innovation, Department of Health and Social Care (England) and the devolved administrations, and leading medical research charities, as well as the Maudsley Charity.

*Acknowledgements:* Professor Louise M Howard, who originally suggested using NLP to identify perinatal self-harm in EHRs. Professor Howard is the primary supervisor of KA's Fellowship.

